# A multitask deep learning approach for pulmonary embolism detection and identification

**DOI:** 10.1038/s41598-022-16976-9

**Published:** 2022-07-29

**Authors:** Xiaotian Ma, Emma C. Ferguson, Xiaoqian Jiang, Sean I. Savitz, Shayan Shams

**Affiliations:** 1 School of Biomedical Informatics, The University of Texas Health Science Center at Houston, Houston, TX USA; 2 Department of Diagnostic and Interventional Imaging, McGovern Medical School, Houston, TX USA; 3 Department of Neurology, McGovern Medical School, Houston, TX USA; 4 Department of Applied Data Science, San José State University, San José, CA USA

**Keywords:** Computational biology and bioinformatics, Image processing, Machine learning, Medical imaging, Computed tomography

## Abstract

Pulmonary embolism (PE) is a blood clot traveling to the lungs and is associated with substantial morbidity and mortality. Therefore, rapid diagnoses and treatments are essential. Chest computed tomographic pulmonary angiogram (CTPA) is the gold standard for PE diagnoses. Deep learning can enhance the radiologists’workflow by identifying PE using CTPA, which helps to prioritize important cases and hasten the diagnoses for at-risk patients. In this study, we propose a two-phase multitask learning method that can recognize the presence of PE and its properties such as the position, whether acute or chronic, and the corresponding right-to-left ventricle diameter (RV/LV) ratio, thereby reducing false-negative diagnoses. Trained on the RSNA-STR Pulmonary Embolism CT Dataset, our model demonstrates promising PE detection performances on the hold-out test set with the window-level AUROC achieving 0.93 and the sensitivity being 0.86 with a specificity of 0.85, which is competitive with the radiologists’sensitivities ranging from 0.67 to 0.87 with specificities of 0.89–0.99. In addition, our model provides interpretability through attention weight heatmaps and gradient-weighted class activation mapping (Grad-CAM). Our proposed deep learning model could predict PE existence and other properties of existing cases, which could be applied to practical assistance for PE diagnosis.

## Introduction

Pulmonary embolism (PE) refers to blood clots in the pulmonary arterial system of the lungs, which usually originate in the deep veins of the legs that break loose and travel to the blood vessels of the lung where they become lodged^1^. PE results in decreased blood flow and oxygen to the lung as well as decreased oxygen levels to other organs in the body^1, 2^. PE is associated with significant morbidity and mortality, and it is the third most common cause of cardiovascular death with an incidence of one case per 1,000 persons in the United States annually^3, 4^. Numerous risk factors predispose patients to the development of PE, including immobilization, recent surgery, history of clotting disorders, malignancy, obesity, pregnancy, cigarette smoking, certain medications such as birth control pills, medical conditions such as heart disease, among others^1, 4, 5^. Early identification and prompt treatment can greatly reduce the risk of death. Thus, accurate diagnosis is crucial in these patients^1, 4–6^.

Computed tomographic pulmonary angiography (CTPA) is currently the most common imaging modality to diagnose pulmonary embolism^7^. The radiologists’sensitivity for detecting PE is reported to range from 0.67 to 0.87, with a specificity ranging from 0.89 to 0.99^8–11^. Deep Learning methods have been developed and showed promising results in detecting PE with a high accuracy, which could further assist radiologists’ decisions^8^. For example, Tajbakhsh et al.^12^ used a 3D convolutional neural network (CNN) with manually extracted features of CT scans called vessel-aligned multi-planar image representation to predict the presence of pulmonary embolism, achieving sensitivity factors predispose of 83% at 2 false positives per volume. Yang et al.^13^ performed a two-stage convolutional neural network with a candidate proposal and a false positive removal subnet. It achieved a sensitivity of 75.4% at two false positives per scan at 0 mm localization error. Instead of using the whole 3D CT scan as an input, Huang et al.^14^ used sliced windows as inputs to 3D CNNs as an end-to-end PE detection solution. This approach reached an area under the receiver operating characteristic curve (AUROC) of 0.84 on the hold-out internal test set and 0.85 on an external dataset for PE detection. Moreover, Huang et al.^15^ proposed a multimodal fusion with deep learning models, combining CTPA image data and electronic medical records. The best model achieved an AUROC of 0.947 on the entire hold-out test set. However, all these studies only considered datasets with a binary classification indicating the existence of PE. The RSNA Pulmonary Embolism CT (RESPECT) dataset^16^ introduced a more challenging problem by containing several study-level labels to predict. Xu’s method^17^ achieved first place in the corresponding Kaggle competition^18^. Their proposed model used a CNN network to extract features for slices and then utilized RNN to process sequences of features for final prediction. However, this solution lacked comprehensive evaluations and had relatively low AUROC scores (Table 1). Suman et al.^19^ also proposed a similar pipeline on the RESPECT dataset and tested their model on a curated external dataset, where the AUROC of positive studies reached 0.949. However, the curated dataset was balanced in positive and negative samples, which was unlikely in real scenarios and publicly unavailable, thus could not be used to compare as a baseline.

In this work, we develop a model that not only detects PE using 3D CTPA images but also predicts the position of PE (left, right, or central), PE condition (acute or chronic), and whether the right-to-left ventricle diameter (RV/LV) ratio is greater or less than 1 in a specific CT image (RV/LV ratio $\ge 1$ suggests the presence of right heart strain) using the RESPECT dataset. Our proposed model consists of a two-phase pipeline to robustly detect and identify PE position, condition and other properties. The first phase uses a 3D CNN for feature extraction and a temporal convolutional network (TCN)^20^ with attention mechanisms in the second phase to perform sequential learning. First, we split the 3D CT scan image into smaller 3D windows to train a deep 3D CNN model. This can capture local contextual information of 3D windows containing several 2D slices. The second-phase model utilizes the learned features in the first step to learn the PE attribute at a study level, we treat the selected features from 3D windows as a sequence and use TCN for sequential learning. In addition, PE only exists in a small subset of studies in our dataset. We, therefore, utilize attention mechanisms to assign weights for features in a sequence, where higher weights indicate higher probabilities of the existence of PE. We train different attention modules for different attributes of PE, since each attribute may focus on different subsets of the whole scan. Besides, integration of CNN and the attention layer introduces interpretability to our model by highlighting the specific region that our model focuses on for prediction. The results of the two phases are reported in the “Results” section, and the classification performances, interpretation outcomes, limitations, and contributions are discussed in the “Discussion” section. The details of our dataset, model, and implementation are described in the “Methods” section.

## Results

Since the data are imbalanced, it would be improper to evaluate the model by prediction accuracy. Thus, we draw the receiver operating characteristic (ROC) curves for each study-level label and compute the AUROC as well. In addition, we produce and examine the sensitivity vs. specificity plots to determine the thresholds of positivity for nine study-level labels. Besides the study-level results obtained after the whole pipeline, ROC curves, AUROC scores, and sensitivity vs. specificity plots are used to evaluate the second-phase training that classifies 3D windows for the window-level label and studies for the nine study-level labels.

### First phase: feature extraction

**Figure 1:**
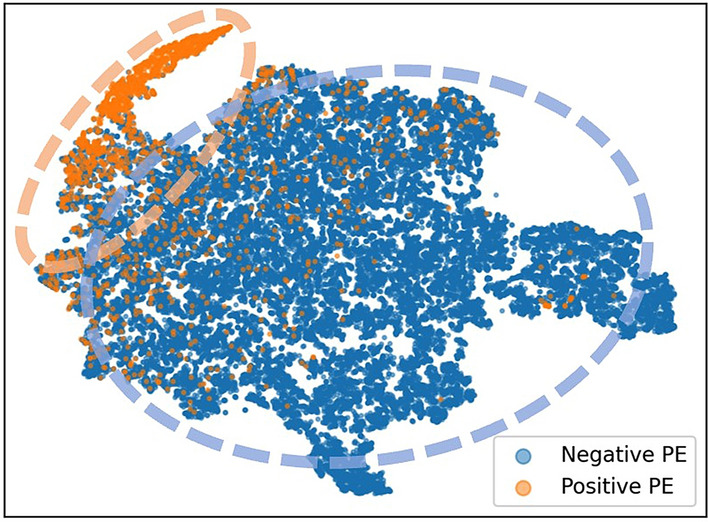
t-SNE plot for all the extracted features of the training set from the first-phase training. The features are extracted from the fine-tuned 3D ResNet-18 model and are 512-d vectors, which are then embedded in the 2D space by t-SNE for visualization. The two groups of positive (orange) and negative (blue) samples are separated well in the 2D space.

The target of the first-phase training is to learn the features for 3D CT scan windows that will be used as inputs for the second phase. Therefore, the learned features from the test samples, extracted from the penultimate layers, are visualized in a 2D embedding space by t-distributed stochastic neighbor embedding (t-SNE)^21^. The two classes are separated well as visualized in Fig. 1. We further analyze the separation by Manhattan distances in the 512-d feature space. We calculate the means for positive samples and negative samples in the 512-d space, denoted as CP and CN respectively. The average Manhattan distance from all the positive samples to CP is 70.15, and that from all the negative samples to CN is 75.70. The distance between CP and CN is 97.21, which indicates that the positive and negative samples are separated well in the 512-d feature space. These well-separated and distinctive clusters among embedded features obtained in the first phase indicate the high quality of feature selection and information embedding in the first-phase model. Therefore, these features could be utilized further in the downstream second-phase training. In addition, the ARUOC on the test set for window-level classification of the first-phase training is 0.9134.

### Second phase: fine-grained classification

The second-phase learning takes the features of 3D windows of a CT image as inputs and predicts the window-level labels and the study-level labels, providing the final prediction of the PE detection task.

#### Prediction performance indicated by ROC curves and AUROCs

**Figure 2:**
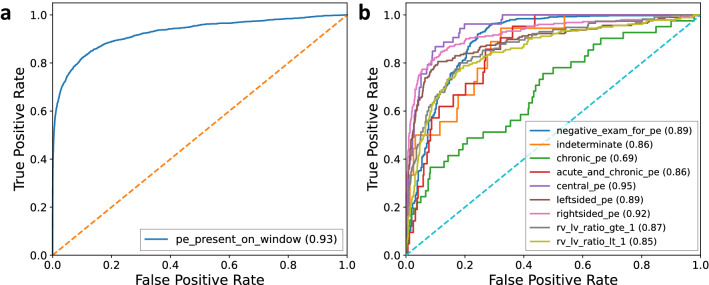
Plots of ROC curves. ROC curves for the window-level (**a**) and nine study-level (**b**) predictions on the test set in the second phase. The values of AUROCs are reported in the parentheses.

The ROC curves of the window-level label and nine study-level labels on the test set for the second training are shown in Fig. 2. The AUROC on the test set for the window-level label increases from 0.9134 of the first-phase training to 0.9258 after the second-phase training. Most of the study-level labels are predicted with AUROCs above 0.85. The AUROCs for central PE and right PE are 0.9477 and 0.9233, respectively. This high performance demonstrates that the model has a great advantage in not only predicting the existence of PE, but also the properties of PE. Table 1 shows the classification performance comparison using AUROC on the same hold-out test set between our model and two previous approaches: (1) Xu’s method^17^ in terms of the window-level label and nine study-level labels; and (2) PENet^14^ in terms of the window-level label and one study-level label indicating negative for PE. Both models are re-trained using the same training and validation set as ours. The comparison shows that we outperformed their results for all labels by a large margin.

**Table 1:** AUROC results on the test set with 95% DeLong confidence intervals. Our method is compared with Xu’s method and PENet on the same train-validation-test split of the RESPECT dataset.

Metric: AUROC	Ours (TCN+Attention)	Xu’s^17^	PENet^14^
PE Present*	0.9258 (0.9183–0.9333)	0.8052 (0.8022–0.8082)	0.8547 (0.8473–0.8622)
Negative for PE	0.8936 (0.8693–0.9180)	0.5923 (0.5546–0.6301)	0.7452 (0.7116–0.7787)
Indeterminate	0.8619 (0.7901–0.9337)	0.7440 (0.6375–0.8505)	–
Chronic	0.6866 (0.5995–0.7737)	0.6479 (0.5649–0.7308)	–
Acute & Chronic	0.8580 (0.8012–0.9149)	0.5876 (0.4710–0.7041)	–
Central PE	0.9477 (0.9263–0.9692)	0.6262 (0.5486–0.7038)	–
Left PE	0.8918 (0.8622–0.9214)	0.5739 (0.5290–0.6188)	–
Right PE	0.9233 (0.9009–0.9457)	0.6054 (0.5645–0.6463)	–
RV/LV$>=$1	0.8708 (0.8346–0.9070)	0.5922 (0.5383–0.6463)	–
RV/VL<1	0.8511 (0.8176–0.8846)	0.5570 (0.5102–0.6039)	–

*Different settings. Ours and PENet are window-level results, while Xu’s is image-level.

#### Sensitivities and specificities

**Figure 3:**
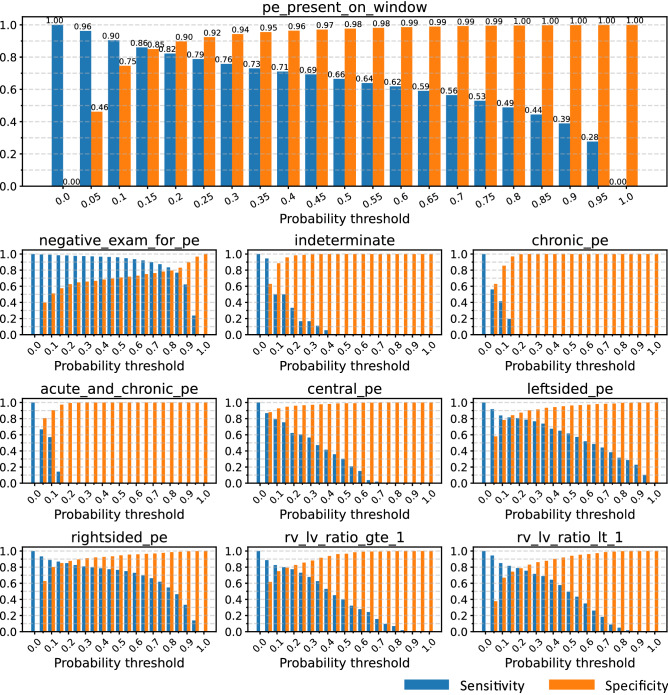
Sensitivity vs. specificity plots. Sensitivity (blue) vs. specificity (orange) for the window-level label (‘pe_present_on_window’on the top indicating whether the PE is present in a certain window) and nine study-level labels over the test set.

Figure 3 shows the sensitivity vs. specificity plots of the test set on the window-level label (whether PE exists) and nine study-level labels. The sensitivities and specificities are calculated as follows:

$$\begin{aligned} \mathrm {sensitivity}&= \frac{\mathrm {number \ of \ true \ positives}}{\mathrm {total \ number \ of \ positive \ smaples \ in \ the \ dataset}} = \frac{\mathrm {number \ of \ true \ positives}}{\mathrm {number \ of \ true \ positives} + \mathrm {number \ of \ false \ negatives}} \\ \mathrm {specificity}&= \frac{\mathrm {number \ of \ true \ negatives}}{\mathrm {total \ number \ of \ negative \ smaples \ in \ the \ dataset}} = \frac{\mathrm {number \ of \ true \ negatives}}{\mathrm {number \ of \ true \ negatives} + \mathrm {number \ of \ false \ positives}} \end{aligned}$$

We select the probability thresholds according to the sensitivity vs. specificity plots of the validation set, and the selected thresholds aim to maximize both sensitivity and specificity. For example, the probability threshold of 0.15 for left PE results in a sensitivity of 0.81 and a specificity of 0.86. The thresholds for the nine study-level labels are reported in Table 2. We can see in Fig. 3 that the sensitivity of detecting PE from a window level reaches 0.86 with a specificity of 0.85, and 0.82 with a specificity of 0.90 on our test set, which is competitive with the radiologists’sensitivity for detecting PE ranging from 0.67 to 0.87 with a specificity of 0.89 to 0.99 generally^8^.

## Discussion

Our proposed two-phase deep learning model for PE detection and identification of its properties could be a helpful tool for radiologists. This method can help to predict the presence of PE, highlighting regions of interest with varying degrees of certainty so that the patient receives a faster and more accurate diagnosis. This tool can help to identify life-threatening PEs, specifically those that are central and acute. It is essential to identify these PEs early since they are associated with higher mortality. This tool can also help rule out PE and different subtypes of existing PE, thereby allowing radiologists to prioritize studies and triage patient care appropriately.

**Figure 4:**
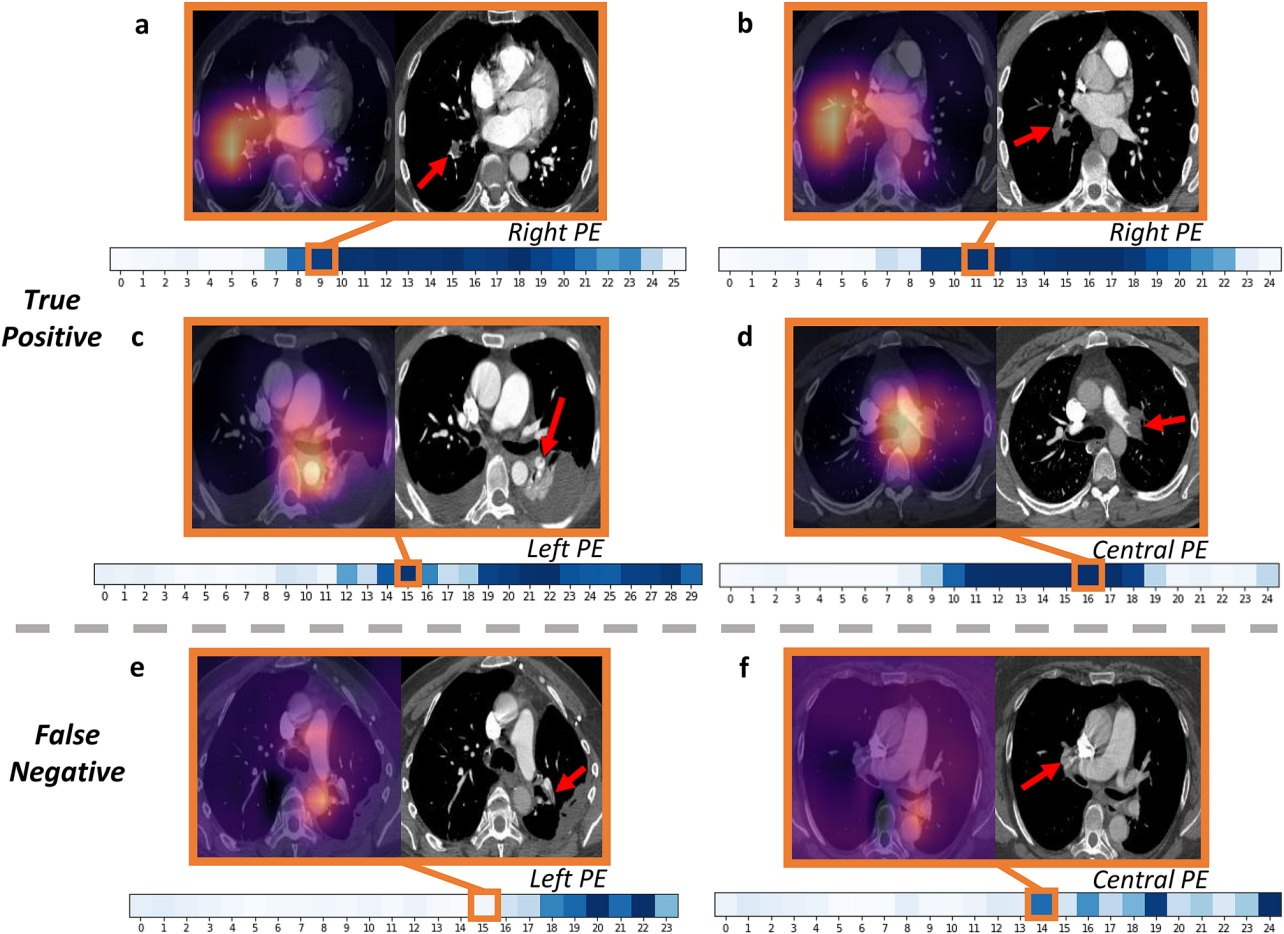
Interpretation with Grad-CAM and attention weights. True positive (**a**–**d**) and false negative (**e**,**f**) samples of Grad-CAM and original image for positional labels. For each sample, the processed CT image (right) and the corresponding attention-mapped image are paired (left). The red arrow points to the precise location of the PE identified by an experienced radiologist. The heatmap below shows the attention weights of all windows in the study containing the image above, while the orange square marks the exact window that includes the image. Darker colors in the heatmap illustrate larger attention weights.

To introduce interpretability and highlight the features selected by the model during prediction, we use Grad-CAM^22^. Here, we focus on the position of the present PE and show selected samples of the true-positive and false-negative results according to the probability thresholds determined in Table 2. Figure 4 illustrates the selected Grad-CAM images and original images for the three positional labels: central, left, and right PE. In addition to the Grad-CAM visualization, we also show the attention weights as the saliency map over the sequences of 3D windows, where darker colors indicate higher attention weights.

**Table 2:** Thresholds for the window-level and nine study-level labels and the correspondent sensitivity and specificity on the validation set.

	Probability threshold	Sensitivity	Specificity
PE Present*	0.15	0.86	0.85
Negative for PE	0.80	0.81	0.80
Indeterminate	0.05	0.93	0.59
Chronic	0.05	0.62	0.63
Acute & Chronic	0.05	0.80	0.80
Central PE	0.05	0.87	0.87
Left PE	0.15	0.81	0.86
Right PE	0.15	0.84	0.82
RV/LV$>=$1	0.15	0.87	0.82
RV/LV<1	0.15	0.73	0.70

*Window-level existence.

Figure 4a–d show the true positive samples. This demonstrates that the model could locate the position of a PE properly. Figure 4a,b are the right lower lobe sub-segmental PEs. The PE in Fig. 4a is located in a peripheral pulmonary artery branch. It is centrally located in the artery and almost completely occludes the blood vessel, indicating that it is acute. The PE in Fig. 4b is also acute, and it occludes the right lower lobe pulmonary artery branch. Figure 4c indicates a tiny, left lower lobe pulmonary embolism in a peripheral branch that is acute and does not occlude the vessel. Figure 4d shows a large PE within the left main pulmonary artery, which is acute and occludes the blood vessel. This kind of centrally located PEs is associated with a higher mortality rate. The attention maps of all the examples above show that the attention weights over the corresponding windows are high, leading our model to pay more attention to those windows with PE present. Some of the selected windows may not have the exact highest attention weight in the sequence, which seems to be a “shift”, but they are generally much higher than the vicinity and are very close to the highest weights if not the same. Also, one sequence could have more than one window showing defects, and we only selected one of the windows to illustrate the details, because it involves great human labor to annotate the actual defects, and we could only select a small subset of the images for annotation instead of annotating all of them. Although our model achieves promising results, there are still many false-negative samples that we need to inspect. Figure 4e,f are two examples of false-negative predictions. Our model fails to detect a small left lower lobe segmental and sub-segmental PE located in Fig. 4e. It does not occlude the blood vessel, and contrast passes around it. In addition, the attention map shows that our model fails to pay attention to the corresponding window that contains this image. Figure 4f displays a right upper lobe segmental PE that is acute but not detected by our model. The attention map shows that the attention weight of the corresponding window are not the highest.

This study also has important limitations. The Grad-CAM for interpretability is only performed on the first-phase training, not going through the parameters of the sequential model in the second phase. In addition, the study-level labels are hierarchical, and some labels may be directly determined by others. For example, a study labeled negative for PE should also be labeled negative for left PE. However, in our model, we do not consider the dependency between the labels and the predicted study-level labels could be inconsistent.

In conclusion, our contribution can be summarized as follows:

- Our two-phase method can detect PE and predict several attributes of existing PE at a study level.
- We split each 3D CT scan image into several smaller windows, ensuring that the model learns local contextual information.
- By implementing multitask attention mechanisms before predicting the study-level labels of PE, our proposed model could focus on specific items in a sequence corresponding to certain attributes for a certain label instead of the whole sequence for all the labels.
- We also visualize and interpret our model using gradient-weighted class activation mapping (Grad-CAM)^22^ and label-specific attention heatmaps to provide insight into the modeling process, alleviating the“black-box”problem of deep learning models.

Our proposed deep learning approach could be a useful tool to facilitate radiologists in PE diagnosis. Future works will include designing more efficient 3D CNN to extract informative features, applying better sequential models for the second-phase learning, and solving the hierarchical dependency of the property labels.

## Methods

This section will introduce our method in terms of its architecture (3D CNN in the first phase for feature detection and TCN for classification in the second phase) and various modules (attention mechanism, loss functions, and interpretation methods). Briefly, the first-phase training extracts features for 3D windows, and the second-phase training takes the extracted features from 3D windows as sequential inputs for final prediction.

### Dataset

The utilized dataset is obtained from the Kaggle competition RSNA STR Pulmonary Embolism Detection^16, 18^. There are 7279 studies in total, and each study consists of multiple 2D slices. The number of 2D slices ranges from 63 to 1083 for the whole dataset, but in 80% of studies, the number of 2D slices ranges from 190 to 296. The dataset has both study-level and slice-level labels. Each slice has a label indicating whether there are any forms of PE present in the slice, while each study has another nine labels indicating other aspects of PE at a study level, such as whether the study is negative for PE, the position of PE (left, right, central), the RV/LV ratio (greater or less than 1), whether the PE is acute or chronic, and whether the PE is indeterminate. The whole dataset is split into 1000 validation studies, 1000 test studies, and 5292 train studies. The positive rates for the nine study-level labels on the train, validation, and test set are reported in Table 3.

**Table 3:** Positive rates for nine study-level labels

(%)	Train	Validation	Test
Negative for PE	67.1	68.1	68.7
Indeterminate	2.3	1.5	1.8
Chronic	4.0	3.9	4.1
Acute & Chronic	2.1	1.5	2.1
Central PE	5.6	5.4	5.3
Left PE	21.1	22.4	20.6
Right PE	25.9	25.7	25.2
RV/LV$>=$1	12.9	14.2	11.5
RV/LV<1	17.6	16.2	18.0

### Data processing

**Figure 5:**
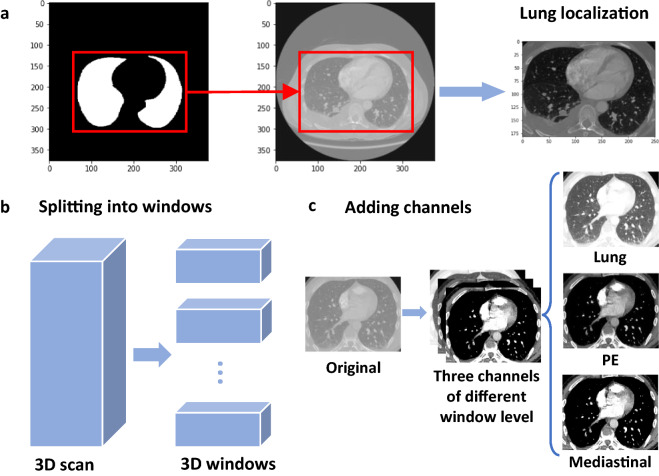
Illustration of data processing. (**a**) Localizing the lung areas according to lung segmentation masks. (**b**) Splitting the whole 3D CT scan into smaller 3D windows. (**c**) Converting single-channel images into 3-channel images.

Figure 5 illustrates the data processing steps. Each study contains an average of 246 slices, and the slices are sorted by the z-axis position from bottom to top to ensure the orders. The raw DICOM pixels are transformed to Hounsfield unit (HU) according to the intercept and slope from the raw data for each study: $\text {HU pixel} = \text {raw pixel} \times \text {slope} + \text {intercept}$. Since each slice has a different thickness, we also resample the slices in each study to ensure the thicknesses are in the same magnitude, i.e., 1 mm for all three dimensions. The labels are also resampled in the same way as the corresponding slices. In addition, lung segmentation^23^ is performed on each slice to decrease the noise, and then we localize the lung area in each study by a 3D bounding box derived from the segmentation masks (Fig. 5a). The labels are also truncated, and only those for the slices inside the bounding box remain.

Furthermore, we treat the processed slices in each study as a 3D image and then split the whole image into several small 3D windows (Fig. 5b). Each window contains 10 slices, and the shape is thus $10 \times H \times W$, where *H* and *W* are the height and width of the slices, respectively. We transform each 3D window into three channels by clipping the HU pixel according to different window levels (Fig. 1c)^24^. In practice, we set the window level (*L*) and window width (*W*) tuples (*L*, *W*) of the boundaries to be $(-600, 1500)$ (lung), (100, 700) (PE), and (40, 400) (mediastinal)^6^. The upper and lower boundaries for clipping the image are $L \pm W / 2$ respectively, which are $(-1350, 150)$, $(-250, 450)$, and $(-160, 240)$. The clipped image is then normalized to the range of [0, 1].

### First phase: 3D CNN

**Figure 6:**
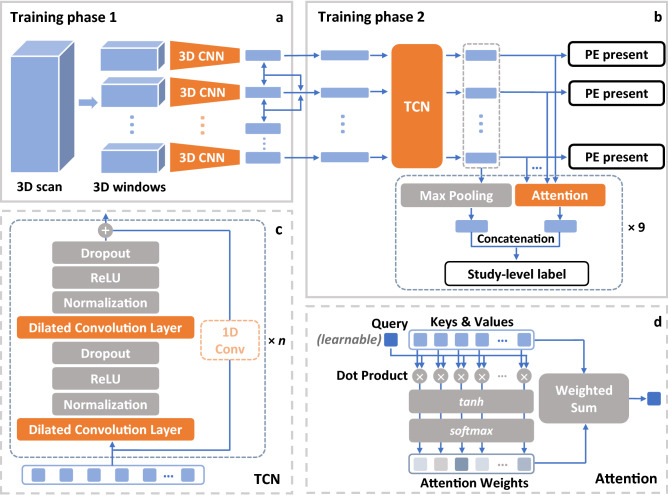
Illustration of the overall pipeline. (**a**) First-phase training framework to extract features. (**b**) Second-phase sequential training architecture. (**c**) Details of temporal neural network (TCN)^20^ in training phase 2. (**d**) Attention mechanism used in training phase 2.

Figure 6 shows the overall pipeline that we used for training. The model in the first phase is a 3D CNN extracting features from 3D windows of pre-processed image slices (Fig. 6a). The average number of split 3D windows in each study is 32. If one of the slices in a window has PE on it, the label for the window is 1, otherwise 0. The purpose of the first-phase training is to learn features that could capture useful information from the 3D windows and then send them into the second phase for further training. As a result, we use the pre-trained 3D ResNet-18 model^25^ provided by PyTorch^26^. The 3D ResNet-18 model, which is a simple and effective backbone for classifying videos or 3D images and is efficient to implement, meets the purpose of the first phase well to extract informative features (as shown in Fig. 1). We then fine-tune the pretrained 3D ResNet-18 model by replacing the output of the last layer with a scalar to fit our binary labels and retraining the model initialized by pretrained weights using the 3D image windows (i.e., a cubic extracted from the original image) of our own dataset. After training, we extract the outputs of penultimate layers as learned features for the 3D windows, which are treated as inputs to the second phase of the model for sequential learning. The size of the extracted feature is 512 which is pre-defined in the 3D ResNet-18 architecture. As a result, we get a 512-d feature for each 3D window. More implementation information is reported in the “Implementation details” section.

#### Loss function

The loss function for the first phase is the binary cross-entropy loss. For each 3D window $\mathbf {I} \in \mathbb {R}^{C \times D \times H \times W}$, the output logit is thus $z \in \mathbb {R}$, and the ground truth label is $y \in \{0, 1\}$. The loss for window *i* can be described as

$$\begin{aligned} l(z_i, y_i) = - [y \cdot \log \sigma (z_i) + (1 - y_i) \cdot \log (1 - \sigma (z_i))] \end{aligned}$$

where $\sigma (\cdot )$ is the sigmoid function. For each mini-batch of size *M*, the loss function is then defined as

$$\begin{aligned} L_{\mathrm {1st}} = \frac{1}{m} \sum ^M_{i=1} l(z_i, y_i) \end{aligned}$$

### Second phase: sequential model with attention

The second phase (Fig. 6b) of training uses the features extracted in phase 1 from 3D windows of a CT image in each study as sequential inputs. The sequence length is set to 40. If a sequence has less than 40 elements, zeros are padded to the end of the sequence; otherwise, the sequence is resized to the length of 40. To extract more information from the context, we subtract each feature of a certain 3D window from the features of its two neighbors and use these two differences as additional inputs by concatenating them to the original feature to form a new 1536-d feature. Then the sequences of new features are sent to a TCN^20^ to capture the overall sequential information. Afterward, the outputs of TCN are used for slice-level prediction, i.e., predicting whether there exists PE on each slice. Meanwhile, nine attention heads are attached to the outputs of the TCN for the downstream prediction of nine study-level labels. Finally, the sum of the outputs of TCN weighted by the attention scores is treated as final embeddings, and we predict each of the nine study-level labels by a two-layer multilayer perceptron (MLP). More implementation information is reported in the “Implementation details” section.

#### Temporal convolutional neural network (TCN)

The basic residual block of TCN is shown in Fig. 6c. It uses dilated convolutional layers to increase the receptive field. In addition, the inputs of the block are added to the outputs as the original residual block^27^. This allows the block to learn residuals to the identical mapping instead of the entire transformation, which could ensure the stabilization of deeper networks and increase the expressive power^20, 27^. Each block consists of two dilated convolution layers with dilation *d* and kernel size *k*, and each layer is followed by weight normalization, ReLU activation, and dropout. If the output and the residual input have different dimensions, a convolution layer with a kernel size equal to 1 will be added to ensure the dimensions are the same when adding. Then the basic residual block is stacked by *n* levels, and for the *i*-th level’s block, the dilation is set to be $d=2^i$. In our implementation, the kernel size is $k=3$, the number of levels is $n=2$, and the dropout ratio is 0.2. The padding for each convolution operation is $(k-1) \cdot d$ and the stride is 1 to make sure the output has the same sequence length as the input. The number of output channels is 128. After transpose, we get 128-d embeddings from the input 1536-d features. The embeddings are either used to classify window-level labels or are sent into several attention mechanisms to obtain study-level predictions.

#### Attention mechanism

The input feature sequences are treated as both keys and values in the attention mechanism (Fig. 6d). We denote the feature sequence after the TCN as a matrix $\mathbf {X} \in \mathbb {R}^{n \times d}$, where *n* is the length of the sequence, and *d* is the dimension of each feature in the sequence (in this case, $n=40$ and $d=128$). We set the query vector $\mathbf {w}_q \in \mathbb {R}^d$ as a parameter to learn through the training, which has the same dimension of each feature. The attention weights $\mathbf {a} \in \mathbb {R}^n$ are obtained from the activation of dot product of keys and queries, and the outputs $\mathbf {e} \in \mathbb {R}^d$ are the weighted sums of the inputs by attention weights on the sequence level:

$$\begin{aligned} \mathbf {a}&= \mathrm {softmax}(\tanh (\mathbf {X} \cdot \mathbf {w}_q)) \\ \mathbf {e}&= \mathbf {a} \cdot \mathbf {X} \end{aligned}$$

In the “Visualization and interpretation” section, we also use the attention weights to illustrate the importance of each 3D window that contributes to a certain output label.

#### Loss function

The loss function for each predicted label is the binary cross-entropy loss as used in the first phase. Suppose in a mini-batch containing *M* studies and each study containing $N_i$ windows, $z_{ij} \, (j = 1,2,\ldots ,N_i)$ denotes the output logit for the window *j* in study *i*, and $z_{ik} \, (k = 1,2,\ldots ,9)$ denotes the logit for the study-level label *k* in study *i*. The window-level loss for the 3D window *j* in study *i* is defined as

$$\begin{aligned} l_{\mathrm {window}}(z_{ij},y_{ij}) = - w \cdot q_i \cdot [y_{ij}\cdot \log \sigma (z_{ij}) + (1 - y_{ij}) \cdot \log (1 - \sigma (z_{ij}))] \end{aligned}$$

where *w* is the weight and $q_i$ is the proportion of positive image windows in study *i*. The study-level loss of study *i* for the *k*-th study-level label is

$$\begin{aligned} l_{\mathrm {study}}(z_{ik},y_{ik}) = - w_k \cdot [y_{ik} \cdot \log \sigma (z_{ik}) + (1-y_{ik}) \cdot \log (1 - \sigma (z_{ik}))] \end{aligned}$$

where $w_k$ is the weight for the label *k* which is pre-defined in the Kaggle competition’s evaluation method, accounting for the relevant importance of the label^28^. Thus, for a mini-batch containing *M* studies with $N_i$ windows in study *i*, the final loss for a mini-batch is

$$\begin{aligned} L_{\mathrm {2nd}} = \frac{1}{\sum ^M_{i=1} (N_i+9)} \sum ^M_{i=1} [\sum ^{N_i}_{j=1} l_{\mathrm {window}}(z_{ij},y_{ij}) + \sum ^9_{k=1} l_{\mathrm {study}}(z_{ik},y_{ik})] \end{aligned}$$

### Visualization and interpretation

We first capture the attention weights on 3D windows for each of the nine labels. This helps us focus on specific features in the sequences and thus directs us to the original 3D window corresponding to those features. Furthermore, we use Grad-CAM^22^ to visually explain the 3D CNN with those selected windows as inputs. Grad-Cam is a localization technique for CNN-based networks, which computes the gradients flowing into the final convolutional layer from a certain target to output heatmaps that highlight specific areas of interest. These areas of interest could be interpreted as the important regions in an image which the network focuses on to predict the target.

### Implementation details

**Figure 7:**
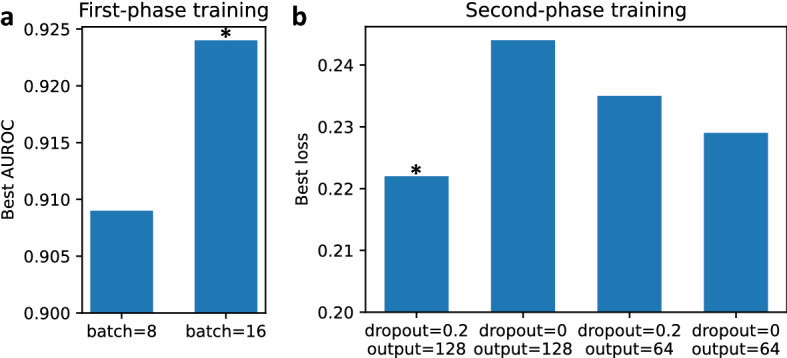
Bar charts of hyperparameter tuning. (**a**) First phase. We tune the batch size (8 and 16) based on AUROC and the optimal one is 16. (**b**) Second phase. We tune the dropout ratio (0 and 0.2) and the number of output channels in TCN (64 and 128) based on loss value. The optimal combination is that the dropout ratio is 0.2 and the number of output channels in TCN is 128.

The two-phase models are trained and tested using a single NVIDIA A100 Tensor Core GPU. The optimizer used for the first-phase training is Adam with a learning rate of 0.0004, and the batch size is 16 selected by hyperparameter tuning (Fig. 7a). We train the model 20 epochs and select the one with the maximum AUROC on the validation set among all the AUROCs of 20 epochs for testing. We also resize the original 2D image slices to $224 \times 224$ and then apply 3D random crop and $15^{\circ }$ rotation to the 3D windows to get $10 \times 192\times 192$ 3D images when training, while we directly resize each 2D image slices to $192 \times 192$ for validation and testing. For the second-phase training, we use Adam optimizer with an initial learning rate 0.0005 decayed every 20 training steps by a multiplicative factor 0.9. The batch size is 64 and the number of epochs for training is 200. We select the dropout ratio as 0.2 and the number of output channels in TCN as 128 by hyperparameter tuning (Fig. 7b). The model with the minimum loss value on the validation set among all the losses of 200 epochs is selected for testing. To select the best model on validation set, we compare the loss in each epoch with the minimum one in all previous epochs, and save the model if the loss in the current epoch is less than the previous minimum one, instead of using early stopping or performing manually via observation.

### Statistical analysis

The evaluation of the first-phase training includes the t-SNE analysis of the extracted features from the training set, and the evaluation of the second-phase training includes the AUROC, sensitivity, and specificity. The 95% DeLong confidence intervals for AUROCs are calculated to measure the variability. We also draw the plots of ROC and sensitivity vs. specificity for all the labels on the test set to better display our results. In addition, the probability thresholds for predicting positive samples are determined by the sensitivities and specificities on the validation set, which ensure high sensitivities while keeping reasonable specificities.

## Data availability

The data that support the findings of this study are obtained from Radiological Society of North America (RSNA) and are publicly available from RSNA STR Pulmonary Embolism Detection Kaggle competition (https://www.kaggle.com/competitions/rsna-str-pulmonary-embolism-detection/data). We also acquired institutional review board approval under protocol HSC-SBMI-13-0549. Since it is a public dataset, we do not need to go through ethical approval.
